# Supplementary material for: Reuniting and Endolymphatic Duct Macrophages: Localization and Possible Roles
Source: Audiol Res. 2025 Nov 20;15(6):160. doi: 10.3390/audiolres15060160 (PMC12641673; doi:10.3390/audiolres15060160)

Supplemental data

1-FIJI Script for masking

```
===========================================================================
run("Open..."); // Open file

run("Convert to Mask", "method=Default background=Default calculate black"); // Convert to mask

// Add mask as a ROI selection and save the ROIset
for (a=1; a<=nSlices; a++) {
	setSlice(a);
	run("Create Selection");
roiManager("add");
}
roiManager("deselect");
pathToROI = getDir("Where do you want to save the ROI set?");
roiManager("Save",pathToROI+"RoiSet.zip");

// Dilate ROIs and save the new ROIset
b = roiManager("count");
for (c = 0; c<b; c++) {
	roiManager("select", c);
	run("Enlarge...","enlarge=-25");
roiManager("Update");
}
pathToEnlROI = getDir("Where do you want to save the enlarged ROI set?");
roiManager("Save",pathToEnlROI+"EnlRoiSet.zip");

// Use the RoiSet just created to keep only the region of interests
// and delete the other parts.
d = roiManager("count");
for (e=0; e<d; e++) {
	roiManager("select",e);
	run("Make Inverse");
	setBackgroundColor(0, 0, 0);
	run("Clear","slice");
}

for (i = 1; i < 128; i++) {
	setSlice(i);
	run("Select All");
	setBackgroundColor(0, 0, 0);
	run("Clear","slice");
}

for (i =342; i < 651; i++) {
	setSlice(i);
	run("Select All");
	setBackgroundColor(0, 0, 0);
	run("Clear","slice");
}
===========================================================================
```

2-MATLAB script for measuring volume, radius and solidity

```
=====================================================================
% STEP 1
% READ TIFF VOLUME IMAGE
macrophages_volume = tiffreadVolume("MASK_macro_ED.tif");
%figure, volshow(macrophages_volume);

% BINARIZE THE IMAGE (MATLAB NEEDS)
bin_vol_macrophages = imbinarize(macrophages_volume);
%figure, volshow(bin_vol_macrophages);

% STEP 2
% CREATE AN IMAGE WITH LABELED COMPONENTS WHO HAVE A CONNECTIVITY
OF 26.
[labeled_macrophages, number_of_macrophages] = bwlabeln(bin_vol_macrophages, 26);
% Print the number of elements
number_of_macrophages

% STEP 3
% COMPUTE STATISTICS ABOUT THE ELEMENTS
macrophages_stats =
regionprops3(labeled_macrophages,"BoundingBox","ConvexImage","Volume", "ConvexHull",
"Solidity");

% STEP 4
% REMOVE ELEMENTS WITH LESS THAN A CERTAIN A VOLUME. SET
volume_threshold % BEFORE RUNNING THE CELL
volume_threshold = 100;
[cleaned_macrophages, number_of_cleaned_macrophages] =
bwlabeln(bwareaopen(bin_vol_macrophages, volume_threshold), 26);
number_of_cleaned_macrophages
stats = regionprops3(cleaned_macrophages);
%figure, labelvolshow(cleaned_macrophages);

macrophage_stats_all = regionprops3(cleaned_macrophages, "Volume", "Solidity");
labels = (1:number_of_cleaned_macrophages)';
macrophage_table_stats = table(macrophage_stats_all.Volume, ...
                    macrophage_stats_all.Solidity, ...
                    labels, ...
                    'VariableNames', {'Volume', 'Solidity', 'Label'});

% STEP 5
% LOOK FOR OUTLIERS AND REMOVE THEM
outlier_macrophages = isoutlier(macrophage_table_stats);
macrophages_stats_no_outliers = rmoutliers(macrophage_table_stats);
% The number of macrophages corresponds to the number of rows in
% macrophages_stats_no_outliers

% MAKE SOME CHANGES IN THE TYPE OF VARIABLE (FROM TABLE TO
ARRAY/CELL).
```

```
macrophages_stats_volumes_voxels = table2array(macrophages_stats_no_outliers(:,1));
% Volume is multiplied per the cube of measure to obtain the values in um3.
volume_measure = 1.6^3; % the first number represents the voxel sixe.
macrophages_stats_volumes_microns = macrophages_stats_volumes_voxels * volume_measure;
macrophages_stats_solidity = table2array(macrophages_stats_no_outliers(:,2));
macrophages_stats_labels = table2cell(macrophages_stats_no_outliers(:,3));

% MEASURE MEAN, MEDIAN AND STANDARD DEVIATION
% Solidity
mean_solidity = mean(macrophages_stats_solidity)
stdev_solidity = std(macrophages_stats_solidity)
median_solidity = median(macrophages_stats_solidity)

% Volume
mean_volume = mean(macrophages_stats_volumes_microns)
stdev_volume = std(macrophages_stats_volumes_microns)
median_volume = median(macrophages_stats_volumes_microns)

% Radius (if considering macrophages as spherical (Vs = (4/3) * pi * r^3))
denominator = 4 * pi;
macrophages_radius_microns = ((3 * macrophages_stats_volumes_microns) /
(denominator)).^(1/3);
mean_radius = mean(macrophages_radius_microns)
stdev_radius = std(macrophages_radius_microns)
median_radius = median(macrophages_radius_microns)

% PLOT VOLUME ON X AXIS AND SOLIDITY ON Y AXIS. EACH MACROPHAGE HAS ITS
% ASSOCIATED LABEL NUMBER
figure, ts = textscatter(macrophages_stats_volumes_microns, macrophages_stats_solidity,
macrophages_stats_labels,"MarkerSize", 10, "TextDensityPercentage", 100);
xlabel("Volume (um^3)");
ylabel("Solidity");

% PLOT SOLIDITY AND VOLUME AS HISTOGRAM
number_of_bins = int32(sqrt(numel(macrophages_stats_solidity)));
% solidity
solidity_hist = histogram(macrophages_stats_solidity, number_of_bins);
xlabel("Solidity");
ylabel("Number of macrophages per bin");

% volume
volume_hist = histogram(macrophages_stats_volumes_microns, number_of_bins);
xlabel("Volumes (um^3)");
ylabel("Number of macrophages per bin");

% VISUALIZE ONE MACROPHAGE
label_number = 13;
want_to_see_macrophage = bwlabeln(ismember(cleaned_macrophages, label_number));
want_to_see_macrophage_stats =
regionprops3(want_to_see_macrophage,"BoundingBox","ConvexImage","Volume", "ConvexHull",
"Solidity");
```

```
ch_image = want_to_see_macrophage_stats.ConvexImage(1,1);
ch_contents = cell2mat(ch_image);
name_of_ch_figure = 'Convex Hull with cluster (alpha=0,04)';
ch_fig = uifigure(Name = name_of_ch_figure);
ch_viewer = viewer3d(ch_fig);
figure, volshow(ch_contents, Alphamap=0.04, Parent = ch_viewer);
saveas(ch_fig,name_of_ch_figure);

% Overlay the convex hull with the cluster
% Crop the original volume to the convex hull dimensions.
cuboid = [want_to_see_macrophage_stats.BoundingBox(1,1)
want_to_see_macrophage_stats.BoundingBox(1,2)
want_to_see_macrophage_stats.BoundingBox(1,3)...
  want_to_see_macrophage_stats.BoundingBox(1,4)-1
want_to_see_macrophage_stats.BoundingBox(1,5)-1
want_to_see_macrophage_stats.BoundingBox(1,6)-1];
cluster_crop = imcrop3(want_to_see_macrophage, cuboid);
labeled_cluster = bwlabeln(ch_contents);
volumeViewer(cluster_crop,labeled_cluster)
```

==

3-Python script for Kruskal-Wallis and Dunn tests

```
=======================================================================
import pandas as pd
import scipy as sp
import scikit_posthocs as skp
#%% READ CSV
volumes_df = pd.read_csv("Macrophage_volumes.csv")
radius_df = pd.read_csv("Macrophage_radius.csv")
solidity_df = pd.read_csv("Macrophage_solidity.csv")

#%% SEPARATE MACROPHAGE GROUPS

# In volume data
vva_volumes = volumes_df[volumes_df.columns[0]]
es_volumes = volumes_df[volumes_df.columns[1]]
dr_volumes = volumes_df[volumes_df.columns[2]]
vca_volumes = volumes_df[volumes_df.columns[3]]
vaq_volumes = volumes_df[volumes_df.columns[4]]
ed_volumes = volumes_df[volumes_df.columns[5]]

# In radius
vva_radius = radius_df[radius_df.columns[0]]
es_radius = radius_df[radius_df.columns[1]]
dr_radius = radius_df[radius_df.columns[2]]
vca_radius = radius_df[radius_df.columns[3]]
vaq_radius = radius_df[radius_df.columns[4]]
ed_radius = radius_df[radius_df.columns[5]]

# In solidity
vva_solidity = solidity_df[solidity_df.columns[0]]
es_solidity = solidity_df[solidity_df.columns[1]]
dr_solidity = solidity_df[solidity_df.columns[2]]
vca_solidity = solidity_df[solidity_df.columns[3]]
vaq_solidity = solidity_df[solidity_df.columns[4]]
ed_solidity = solidity_df[solidity_df.columns[5]]

#%% COMPUTE KRUSKAL-WILLIS TEST

# For volume data
volumes_kruskalwillis = sp.stats.kruskal(vva_volumes.dropna(),
                                es_volumes.dropna(),
                                dr_volumes.dropna(),
                                #vca_volumes.dropna(),
                                vaq_volumes.dropna(),
                                ed_volumes.dropna())
print('Kruskal-Willis test for volumes: ', volumes_kruskalwillis)

# For radius data
radius_kruskalwillis = sp.stats.kruskal(vva_radius.dropna(),
                                es_radius.dropna(),
                                dr_radius.dropna(),
                                #vca_radius.dropna(),
```

```
                                    vaq_radius.dropna(),
                                    ed_radius.dropna())
print('Kruskal-Willis test for radius: ', radius_kruskalwillis)

# For solidity data
solidity_kruskalwillis = sp.stats.kruskal(#vva_solidity.dropna(),
                                    es_solidity.dropna(),
                                    dr_solidity.dropna(),
                                    vca_solidity.dropna())#,
                                    #vaq_solidity.dropna(),
                                    #ed_solidity.dropna())
print('Kruskal-Willis test for solidity: ', solidity_kruskalwillis)
#%% COMPUTE DUNN'S TEST

# For volumes
volumes_dunn_data = [vva_volumes,
            es_volumes,
            dr_volumes,
            vca_volumes,
            vaq_volumes,
            ed_volumes]

volumes_dunn_test = skp.posthoc_dunn(volumes_dunn_data)
boolean_volumes_dunn_test = volumes_dunn_test < 0.05
#%% For radius
radius_dunn_data = [vva_radius,
            es_radius,
            dr_radius,
            vca_radius,
            vaq_radius,
            ed_radius]

radius_dunn_test = skp.posthoc_dunn(radius_dunn_data)
boolean_radius_dunn_test = radius_dunn_test < 0.05
#%% For solidity
solidity_dunn_data = [vva_solidity,
            es_solidity,
            dr_solidity,
            vca_solidity,
            vaq_solidity,
            ed_solidity]

solidity_dunn_test = skp.posthoc_dunn(solidity_dunn_data)
boolean_solidity_dunn_test = solidity_dunn_test < 0.05
```

==

4- Box plots for macrophage volume, radius and solidity. Red lines indicate median values, bottom and top of each blue box are the 25th and 75th percentiles, whiskers extend to the full range or to 1.5 times the range included in the box. Data falling outside the whisker range are considered outliers and indicated with red crosses. Abbreviations: VVA: vein of the vestibular aqueduct, Esi: Endolymphatic sinus, RD: reuniting duct, VCA: vestibulocochlear artery,
Vaq: vestibular aqueduct, ED: endolymphatic duct.

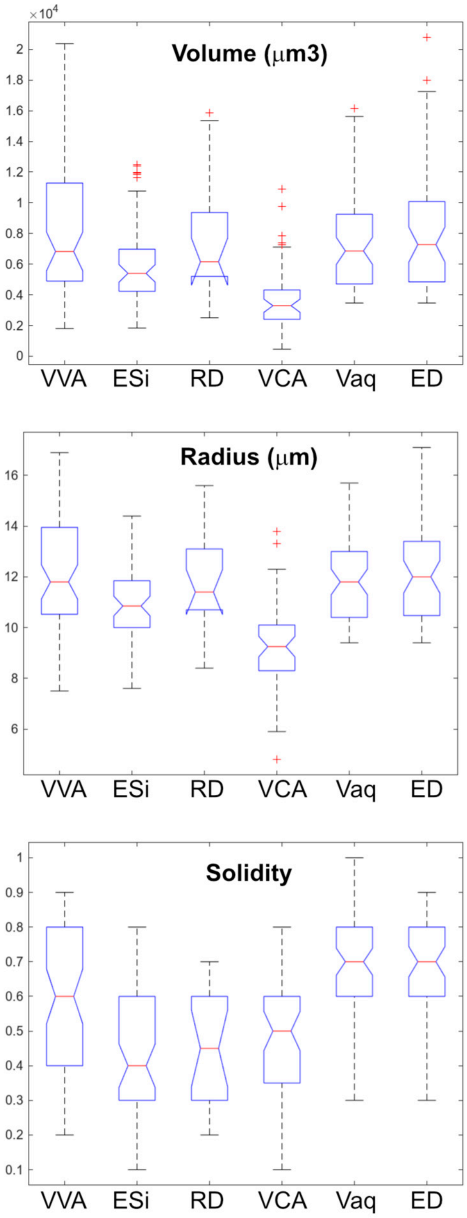

Supplement: Supplementary file 1 [file audiolres-15-00160-s001.zip › audiolres-3836953-supplementary.pdf]
